# Implementation of a Sense of Home in High-Density Multicultural Singapore Nursing Homes: Challenges and Opportunities

**DOI:** 10.3390/ijerph19116557

**Published:** 2022-05-27

**Authors:** Jung-Joo Lee, Tse Pei Ng, Ivan Kurniawan Nasution, Jia Yen Eng, Renee Desneige Christensen, John Chye Fung

**Affiliations:** 1 College of Design and Engineering, National University of Singapore, 4 Architecture Drive, Singapore 117566, Singapore; ngtsepei@gmail.com (T.P.N.); ivan.nst@nus.edu.sg (I.K.N.); akifjc@nus.edu.sg (J.C.F.); 2 Department of Rehabilitation, National University Hospital, 5 Lower Kent Ridge Rd., Singapore 119074, Singapore; jia_yen_eng@nuhs.edu.sg; 3 International WELL Building Institute, 220 5th Avenue, New York, NY 10001, USA; renee.christensen@wellcertified.com

**Keywords:** nursing home, aging, high-density, sense of home, multicultural, culture

## Abstract

Many studies have introduced principles for creating a sense of home in nursing homes, yet they mostly feature cases from low-density developments in Western countries. This raises a question about how those principles are interpreted and implemented in other cultural contexts, especially in high-density, multicultural environments such as Singapore. This paper examines how a sense of home is implemented in Singapore nursing homes, with a specific focus on the role of the built environment. Participant observations were conducted in five nursing homes in Singapore comprising various architectural design typologies, with the focus on the residents’ everyday interactions with their built environment. The study identified the extent of the presence of a sense of home in Singapore’s nursing homes and the prevalence of an institutional care model. More specifically, the study explicates Singapore nursing home residents’ management of privacy and personalization in shared spaces, illuminates the need for holistic implementation of homelike environments integrated with building designs and care programs and reiterates the pivotal role of social relationships in fostering a sense of home for the residents in the nursing homes.

## 1. Introduction

A nursing home has a dual nature as an institution that delivers clinical care and a home that provides collective living arrangements for a good quality of life. However, delivering both quality care and a homelike environment is challenging [1]. Creating a sense of home is recognized as pivotal for the physical and psychological well-being of older residents in a collective living environment. A sense of home is a multifactorial phenomenon [2]. It is highly influenced by social and personal characteristics, the ambient and built environment, architectural design and the culture that defines the role of a nursing home in modern societies. As Fung [3] posits: “Particularly in a 21st century milieu of high-rise and high-density urban living, the conventional model of nursing home is perched on top of a metaphorical sand-pile ready to erupt an avalanche of transformations.” The evolving notion of a sense of home is thus subjected to constant interpretations, re-interpretations and contestations.

Many extant studies have introduced principles and recommendations to foster a sense of home in nursing home environments. However, most were cases perpetuating a Western cultural lens and were contextualized in low-density settings. One can argue that there is a tension between the universality and cultural specificity of how older residents in different cultural contexts do, and ought to, experience a sense of home. For example, in many nursing home studies from Western countries, “home” is often associated with having a private room with an attached bathroom, and a shared bedroom is unacceptable for most residents [4,5]. A few recent non-Western studies provide differing views. Sima’s [6] work on residential facilities in Shanghai and the work by Low et al. [7] in Hong Kong both reported the Chinese residents’ preference for a shared room over a private one, because of their values regarding collectivism and associations with their past lifestyles. One could surmise that these two studies relate specifically to a deep traditional Chinese culture and may not apply to a modern multicultural society such as Singapore, despite the fact that its population predominantly comprises citizens of Chinese ethnicity.

The process of Singapore’s modernization has resulted in significant differences in values, outlook and identity within societies still steeped in the Chinese traditions. While cultural differences have been recognized as important factors in the design of care models and environments—“culturally sensitive care” as Lee [8] puts it—there has been little attention given to how a “sense of home” is interpreted and implemented in different locales and cultures. This is especially pertinent for a multi-ethnic society such as Singapore, whose diverse cultural practices—despite the predominance of an ethnic Chinese population—coalesce in a collective living environment such as a nursing home.

This paper examines how a sense of home is implemented in Singapore nursing homes, in comparison to the known practices in Western countries. Singapore has distinctive environmental and cultural conditions, being a metropolitan city-nation with high-rise and high-density environments, with centralized urban public housing policies where more than 80 percent of its citizens live in public housing flats. Furthermore, it is multicultural, multi-ethnic and multi-religion in its demographics. Reflecting the current shift towards a homelike setting for aged care, Singapore nursing homes have been experimenting with new care models and environmental designs [9] that challenge previously established outcomes and raise the following questions. How are the principles and recommendations for fostering a sense of home in nursing homes interpreted and implemented in Singapore? What are the mismatches and challenges? How do the older residents in Singapore nursing homes experience a sense of home?

To explore these questions, this study employed a participant observation method that delved into the everyday interactions of older residents with their built environments and organizational settings. Findings from five nursing homes in Singapore are discussed against the background of the extant literature regarding a sense of home in aged care facilities. Various interpretations, challenges and opportunities in creating a sense of home in Singapore nursing homes are unpacked.

## 2. Literature Review

### 2.1. Environmental Aspects of a Sense of Home in Nursing Homes

Numerous empirical studies have identified factors for creating a sense of home in nursing homes. They looked at the physical and environmental settings [10,11,12,13,14,15], social relationships [16,17,18] or the quality of care and conduct of everyday routines [5,14,19]. As many authors explain, these factors are not mutually exclusive, yet they influence each other [11].

We conducted an extensive literature review, employing a snowballing method [20] to gain an overview of what constitutes the environmental elements of a sense of home in a nursing home context. From the search results, a list of recommendations commonly found across various studies was extracted (see Table 1). These recommendations are mostly centered around the themes of private spaces, personal belongings, ambience, appropriate levels of stimulation, connection with nature, and social interaction.

The majority of studies on a sense of home in nursing homes come from cultures of European origin such as North Europe (e.g., [2,4,5,11,14,17,18,21,22,23,25,26]), Australia and New Zealand (e.g., [13,27,28]) or North America (e.g., [14,15,16,29,30,31]). However, as a sense of home is an experienced phenomenon subject to individual perception and interpretation [2], one may argue that these studies were grounded in the cultural contexts of the nursing homes and their residents. Therefore, careful consideration is needed when applying the recommended strategies from such studies to other cultural contexts.

Only a few studies have reported different cultural interpretations of the care models in nursing homes outside Europe, North America and Australasia. Some studies discovered that Chinese elders in nursing homes in Hong Kong and Shanghai valued communal living more than privacy and autonomy [6,7]. A few other studies reported cultural discrepancies experienced by South Asian residents in UK nursing homes in terms of human relationships and spirituality [32,33]. Although these studies indicate cultural differences in residents’ experiences, needs and preferences in nursing homes, they were focused on specific topics such as privacy, rather than examining multiple aspects that constitute a sense of home in a holistic manner.

### 2.2. Living Environments in Singapore

Singapore is characterized as a high-density and high-rise urban environment. Since the 1960s, the government has instituted a massive physical transformation of the city from horizontal *kampongs* (Malay word for village) to vertical living in high-rise buildings. Today, over 80% of Singaporeans live in public housing—typically a flat in a multistoried apartment block within an integrated neighborhood developed by the Housing Development Board (HDB). The notion of “home” to Singaporeans is thus closely associated with this typology of public housing and its integrated neighborhood amenities.

Pragmatism is as much a national ideology as it is a shared value instilled in the everyday life of Singaporeans, and it is deeply embedded in the nation’s public housing program. Although sometimes political and problematic [34,35], pragmatism is both a belief and a method that has contributed to the progress of achieving a stable and well-ordered society [36] and to rapidly housing Singaporeans in a high-density environment [37]. One expression of this ideology is the formulaic planning of self-sufficient public housing estates that are integrated with commercial (shops, supermarkets, offices, wet markets, etc.), institutional (schools, places of worship, community centers, etc.) and recreational facilities (sports fields and facilities, town parks and neighborhood parks, precinct gardens, multi-purpose halls, playgrounds, outdoor gyms, etc.) [34,35].

Concurrently with rapid densification and industrialization, Singapore is facing an increasingly aging population. It is projected that a quarter of its population will be 65 years old and above by 2030, and by 2050 about half of the population will be 65 years old and above [38]. Further, the rise of dual-income nuclear families and the concomitant decline of extended multigenerational families, as well as an increase in age-related degenerative disorders due to increased longevity, render it difficult for families to remain the primary caregivers of elders in their own homes. Consequently, despite a long-held sense of filial piety and traditional values common to many Asian cultures, nursing homes have become increasingly relevant as resource- and expertise-challenged families try to cope with the burden of caring for a frail and dependent elderly loved one [9,39].

At present, nursing home beds in Singapore number close to 17,000. Other than infrastructural provision, other challenges for nursing homes include outdated care practices [39], a high dependency on a foreign workforce [9] and negative perceptions of the institutional model of care [40].

## 3. Materials and Methods

### 3.1. Five Nursing Homes in Singapore

The study on the sense of home presented in this paper is part of a larger interdisciplinary research project covering multiple facets of collective aged care in nursing homes. The research team consisted of the disciplines of environmental psychology, medicine, gerontology, architecture, design and occupational therapy. The study in this paper focused on residents’ experiences with built environments and was led by sub-teams consisting of occupational therapists, architectural researchers and design researchers, while other sub-teams consisting of medicine and gerontology researchers examined other aspects such as the residents’ quality of life and their well-being or the reverse [41].

Five nursing homes (hereafter named NH1 to NH5) were chosen as cases for this study. The five nursing homes represented varying typologies, models of care and years of completion. Within the ambit of regulatory guidelines for nursing homes, each nursing home had developed slightly different housing models and care programs, ranging from conventional clinical care to person-centered care.

Most nursing homes in Singapore provide shared bedrooms where at least four residents share a room. Four of the five nursing homes in this study adopted a hospital ward type of design, with an average occupancy of six persons sharing an open bedroom space (see Table 2). NH5 had 2-person bedrooms but they were primarily for the segregation of residents that required special attention.

Apart from NH5, the other nursing homes in this study were housed in buildings higher than five floors. NH2 and NH4 had a conventional ward-type design with multi-bedded rooms opening onto a long corridor leading to shared activity/dining spaces. Such a typology often embodies the practice of housing a large number of residents on one floor and leads to concerns about safety and security above the person’s need for competency in coping with the environment [42]. NH1 was the result of a recent pilot for a “household” typology, with a cluster of four multi-bedded bedrooms (four occupants per bedroom) arranged around a shared living and dining area in each household.

All five nursing homes were operating at or near full capacity, and their resident populations were in line with the ethnic distribution of Singapore’s demographics (76% Chinese, 15% Malay and 7% Indian). Most residents were non-ambulant, either wheelchair- or bed-bound, and required assistance in some activities of daily living (ADL) tasks.

Around 100 residents from each nursing home (17.7 to 52.6 per cent of the total number of residents) were enrolled in the research (see Table 3). The participants were mostly in their 70s and 80s, and almost equal numbers of male and female residents participated. The participants’ ethnicities reflected the overall ethnic distribution of each nursing home. Most participants required physical assistance and were either wheelchair-bound or bed-bound, i.e., Category III or Category IV, according to the Residential Assessment Form (RAF). This also reflects the overall population of the residents as presented in Table 2. Table 3 summarizes the profiles of the participants.

### 3.2. Participant Observation

Data were collected through participant observation and focus group interviews in the five nursing homes over a six-month period in 2018. The observations were built on a naturalistic inquiry [43], aiming to understand the particularities of a phenomenon in its natural setting and from the perspective of those involved.

Multiple sessions of participant observation were individually conducted by four occupational therapists, three architectural researchers and three design researchers. The occupational therapists consisted of one principal therapist with 15 years of training and three senior therapists with five to ten years of training. The three architectural researchers had one to nine years of training, and the three design researchers had one to two years of training. The architectural researchers and design researchers were guided by principal investigators who had 15 to over 30 years of training. Among the ten researchers in total, two researchers were male, and the rest were female. Two architectural researchers were Malay and Tamil, respectively, while the rest of them were Chinese.

This multidisciplinary team of researchers contributed an “insider’s point of view” through ethnographic observation [44] and diverse domain knowledge on care delivery and the built environment. The occupational therapists focused on the impact of the environment on the occupational participation of the residents. The architectural researchers examined the environmental and spatial features and how they shaped the experiences of the residents, care staff and visitors. The design researchers focused on the residents’ everyday social and emotional experiences with the artifacts and the built environment. While the observational data were collected by this group of researchers, the findings were reviewed by the larger interdisciplinary team through project consortium meetings.

During the participant observation, the researchers recorded field notes, photographs, sketches and videos. Researchers asked the caregiving or administrative staff or the residents brief questions when clarifications were needed, taking care not to disrupt normal daily life and caregiving workflows. The observation sessions covered different days of the week (including weekends) and residents’ full-day schedules to capture, as much as possible, the different activities and occurrences in the nursing homes. While written consent from the participants was waived by the Institutional Research Board for this observational study, authorized care staff of the nursing homes explained the research activities to the residents and obtained their verbal consent prior to the observation sessions.

### 3.3. Focus Group Interviews

After participant observations were completed in each nursing home, focus group interviews were conducted to validate the findings of the observations. Two focus group sessions were held for each nursing home: one with four to five residents to validate the researchers’ understanding of the residents’ experiences and another with five care staff to gain a clear picture of institutional and operational factors. Holding separate sessions ensured that each group was free to express its thoughts.

Participants were selected by the nursing home management with purposive sampling using the researchers’ criteria of being able to communicate (for residents) and being involved in the day-to-day care delivery (for staff). Written consent was obtained from the residents with the assistance of the authorized caregivers of the nursing homes. Each interview session was conducted by the four researchers who also conducted participant observations. The principal occupational therapist with over 15 years of training (female, Chinese) and one design researcher with two years of training (female, Chinese) facilitated the session, while the architectural researcher with nine years of training (male, Malay) and one senior occupational therapist with ten years of training (female, Chinese) assisted at each session as a note-taker and an observer. Each interview session lasted 1 to 1.5 h, and the discussions were conducted simultaneously in English and Mandarin. Sessions were audio-recorded and transcribed for analysis.

### 3.4. Data Analysis

**Figure 1:**
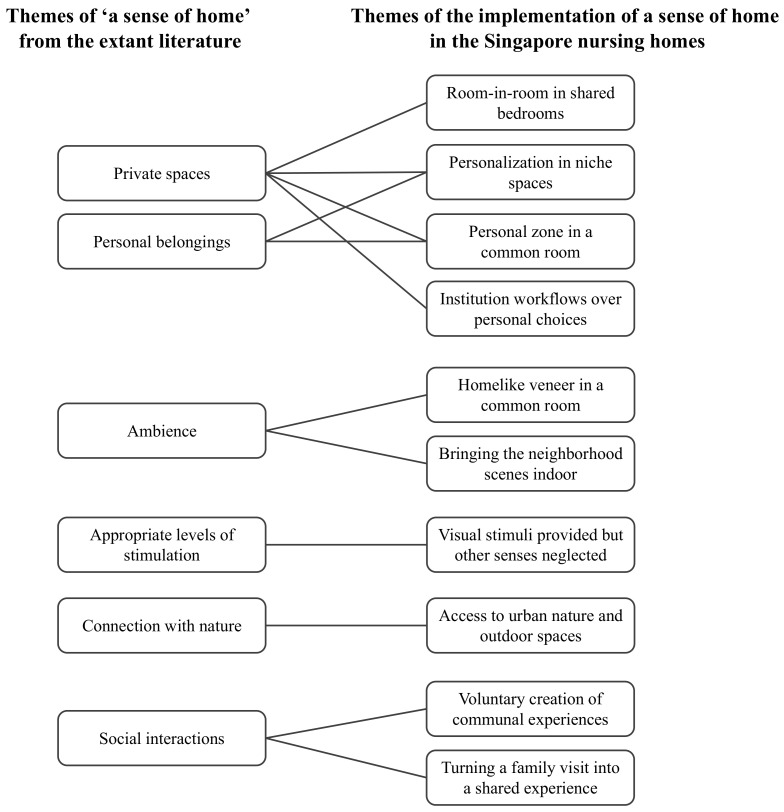
Ten themes relating to the implementation of a sense of home in the Singapore nursing homes, mapped against the themes from the extant literature.

Data analysis was conducted by the multidisciplinary team in several steps. Initially, each disciplinary team conducted a preliminary analysis on its collected data using thematic analysis [45]. Thereafter, two rounds of data synthesis workshops were conducted. Firstly, the findings from the occupational therapists, architectural researchers and design researchers were shared and categorized. In this synthesis process, themes that highlighted the nursing homes’ practices relating to a sense of home emerged. Secondly, the relevant themes were discussed and mapped against the prevailing recommendations for a sense of home as identified through the literature review (Figure 1). This process identified specific local practices or challenges in implementing a sense of home.

## 4. Results

### 4.1. Room-in-Room in Shared Bedrooms

**Figure 2:**
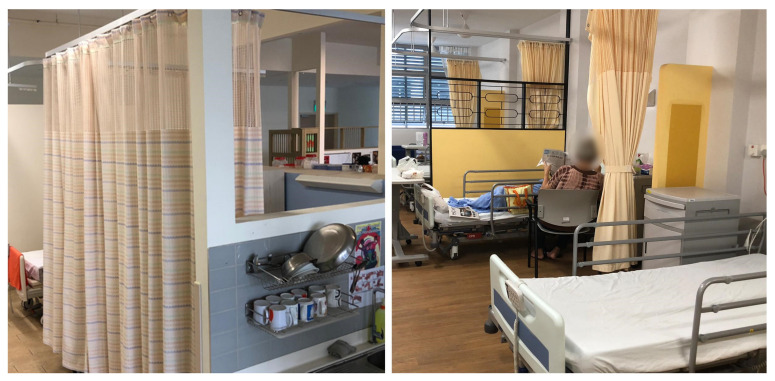
Use of the corner walls, small cabinets and curtains for privacy in shared bedrooms: (**left**) NH1 and (**right**) NH3.

Many extant studies recommend the provision of individual rooms where elderly residents can enjoy private activities and create their own environment with personal belongings. In the five nursing homes in this study, privacy was achieved through a small private corner at the bedside, demarcated by corner walls, furniture or curtains. For example, in the household model of NH1, corner walls enclosing two to three sides of the bedrooms allowed the establishment of personal boundaries, making each bed space look like a “smaller room” (see Figure 2). This implied “room-in-room” is common in many nursing homes. NH3 had parapet walls demarcating the ward’s boundary and partial partitions between beds. These spatial appropriations make the bed spaces less visible from the living room or corridor spaces.

Semi-ambulant or non-ambulant residents, however, did not have autonomy or control over the essential level of privacy in shared bedrooms and relied on caregiving staff to draw the bedside curtains. Often, the caregiving staff did not pay careful attention to ensure that the curtain was fully closed during personal care, and this resulted in an infringement of the resident’s sense of dignity. Free-standing curtains made access even more difficult, as was observed in NH5 when a standing curtain fell over a wheelchair-bound resident trying to get into the bedroom. Standing curtains were mostly used by caregiving staff when conducting personal care tasks such as changing adult disposable undergarments or toileting.

### 4.2. Personalization in Niche Spaces

**Figure 3:**
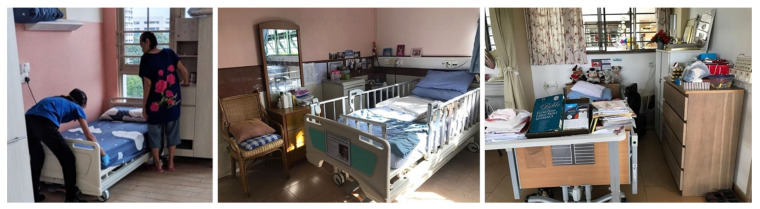
Personalization at the bedside space: (**left**) NH1, (**middle**) NH5 and (**right**) NH4.

Residents claimed such spaces through placement of their personal belongings. Residents in NH1 arranged personal items in their bedside area, such as bedsheets, pillows, soft toys, pictures, drawings, and so on (see Figure 3). NH5 allowed its residents to bring furniture from their own home, making the space more familiar and personal to them. In some cases, residents’ personal items seemed to clutter their bedsides, but they created a sense of homeliness and orderliness. These non-institutional elements increased the sense of familiarity with their past.

“They [staff] want to tidy up, but I say no. I know where my stuff is. I can find where … It feels more home to me.”—A female resident in NH4.

### 4.3. Personal Zone in a Common Room

**Figure 4:**
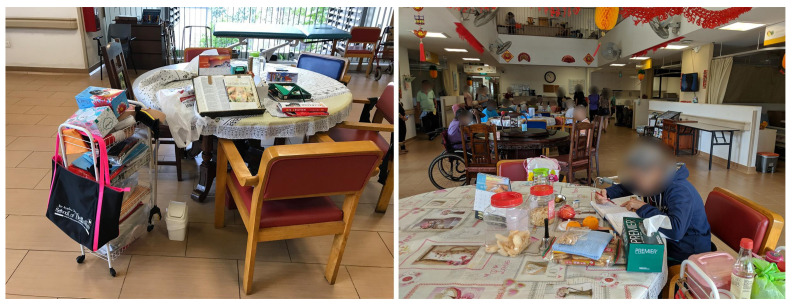
A resident personalizes her regular seat at a dining table in the common space of NH4.

Compensating for the lack of personal space in bedrooms, some residents appropriated spaces in the common rooms to support their personal activities. For example, residents in NH4 claimed seats around a dining table by placing their personal belongings to set up a “personal zone” around them (Figure 4). This behavior resembles the particularly Singaporean practice of *chope*-ing, i.e., reserving a table at a food center using personal belongings. This *chope*-ing is a socially acceptable practice when claiming a personal territory in a shared space in Singapore [46]. While the residents managed to establish territorial boundaries to create their own “personal zone” visually, the excessive noise from the common room interfered with auditory privacy.

### 4.4. Institution Workflows over Personal Choices

**Figure 5:**
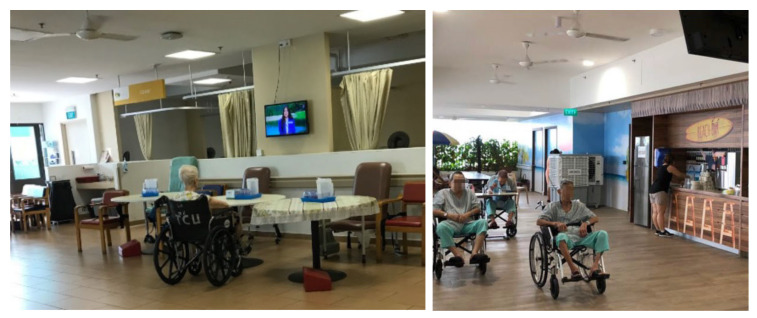
(**Left**) a resident who was moved along with the furniture during the cleaning time in NH4. (**Right**) residents who were moved to the common room for staff monitoring in NH2.

While the residents tried to create personal spaces and develop a sense of privacy, these practices were limited by, or were suspended by, the operational routines of the nursing homes. In NH4, for example, the residents were ushered back to their bedrooms after lunch regardless of their wishes so that the caregiving staff could clear the tables and clean the floor. A resident who wanted to continue spending time in the common area was moved aside along with the furniture, to make way for the cleaning machine (see Figure 5).

In NH2, the residents were put in wheelchairs and gathered in the common area so that the staff could monitor them efficiently. They were not allowed to walk or move by themselves and were left to watch the ceiling-mounted TV, which did not engage most residents because of the high level of ambient noise, disrupting their ability to hear the sounds from the TV. This is especially problematic, as many elderly residents are hearing-impaired. Some residents were observed sitting passively, disassociated from their environment. In NH5, when the staff were busy, residents in wheelchairs were placed against the corridor’s wall, with their wheelchairs strapped to the railing for safety. During the focus group interview, the management and care staff explained that they always need to prioritize the residents’ safety first, in some circumstances over autonomy or a sense of home. Residents during the focus group interview displayed an understanding and acceptance of this practice.

“For me, it’s okay. Because I’m not so fussy about that, because they [staff] have to, you know, find a time for me.”—A female resident in NH4.

### 4.5. Homelike Veneer in a Common Room

**Figure 6:**
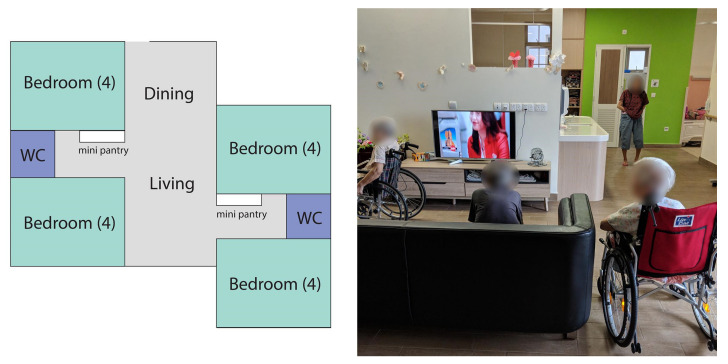
(**Left**) the household layout in NH1 with living and dining space at the center surrounded by four 4-bed bedrooms. (**Right**) a living room in NH1.

In the five nursing homes, the high population density in a living unit invariably makes the environment look and feel more like an institution than a home. Working within the constraints, the nursing homes adopted various strategies to create a homelike ambience. NH1’s household model, where 16 residents live in one household unit, has a floor plan similar to a typical public housing flat in an HDB apartment building in Singapore. In every household unit, four bedrooms were clustered around a living/dining room (Figure 6).

**Figure 7:**
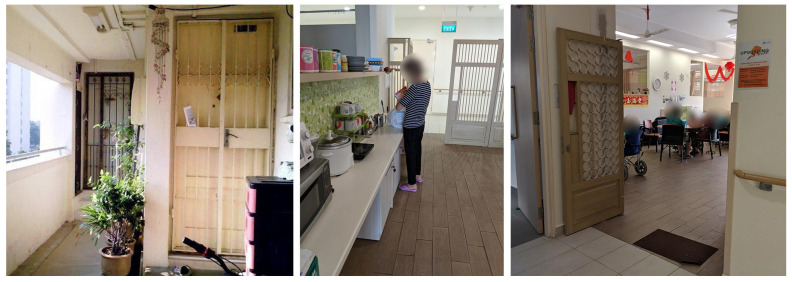
(**Left**) entrances to typical public HDB flats in Singapore. (**Middle**) entrance gate at NH1. (**Right**) entrance gate at NH3.

The nursing homes attempted to create a homelike ambience by decorating the common area with the symbols and aesthetics of HDB designs. In NH1 and NH3, each unit had an entrance gate that resembled those found in HDB housing (Figure 7).

**Figure 8:**
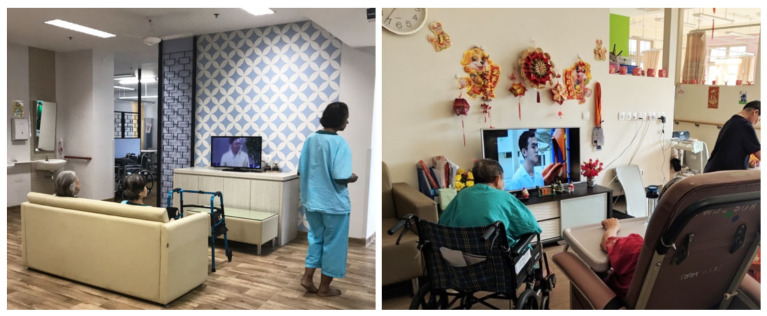
(**Left**) furniture configuration resembling a living room in NH3. (**Right**) homelike decorations in the common area in NH1.

NH3 and NH4 had ward-type layouts similar to a dormitory, with a larger number of residents per living unit (48 residents in NH3 and 54 residents in NH4; see Table 2). The large living unit was segmented into smaller clusters using furniture arranged in a living-room style, e.g., a TV set with a sofa and coffee table. These nursing homes also installed wallpaper depicting past local scenes or iconic elements to evoke a sense of nostalgia. Furthermore, some nursing homes incorporated traditional furniture commonly found in Singaporean homes. In NH1, seasonal festive decorations for traditional Chinese, Malay, Indian and Christian holidays were incorporated (Figure 8). Small ordinary everyday items such as books and potted plants in the living room also helped to create a homelike ambience.

While the nursing homes in this case study attempted to emulate a homelike ambiance through physical layout and visual aesthetics, the engagement of the residents in the home-making activities remained low. Since the residents never participated in the design or decoration of their environment, they missed the opportunity to engage in home-making activities. Furthermore, access to the various everyday household objects was very limited or even prohibited in some instances. Those homelike elements were more like “exhibits” than items provided for everyday interaction. This minimized the ability of the residents to engage in home-making behaviors.

### 4.6. Bringing the Neighborhood Scenes Indoors

**Figure 9:**
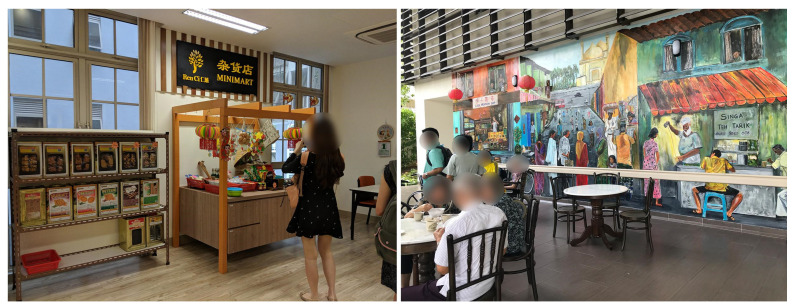
(**Left**) a vintage “mama shop” corner in the NH1 activity space. (**Right**) wall mural and furniture in NH4 that evoke the ambience of a local coffee shop.

The nursing homes in this study allowed the residents very limited access to the outdoors, due to their emphasis on safety and security. Instead, their solution was to bring neighborhood elements inside the nursing home facility through proxy interventions. For example, NH1 designed a space to mimic a vintage local convenience stall, called a “mama shop” (Figure 9), with scheduled opportunities for residents to purchase snacks from the shop. Similarly, NH4 installed a mural next to its main entrance, depicting a scene with itinerant street hawkers of the past, together with tables and chairs, to create a space resembling a common type of Singaporean local coffee shop called a “kopitiam”, and weekly events were organized serving local food and beverages to the residents. Scenes and activities of the outside world were re-created by these nursing homes to encourage residents to engage in familiar activities that are common in the local culture.

Despite efforts to provide residents with opportunities to engage in familiar activities, independent access to such spaces was limited. Due to the nursing homes’ focus on safety and security, the residents could only access these spaces within a rigidly structured schedule. This reduced the positive benefits these spaces could potentially offer in promoting a sense of belonging to the larger community, a continuation of their past life course and a sense of home in the collective setting of a nursing home within the context of Singapore.

### 4.7. Visual Stimuli Provided but Other Senses Neglected

Residents had limited access to and control over sensory stimulation. The sensory stimuli provided were limited to visual stimulations, but consideration of other senses was lacking. During the focus group interviews, residents complained about the constant ringing of the call bells and shouting from other residents.

### 4.8. Access to Urban Nature and Outdoor Spaces

**Figure 10:**
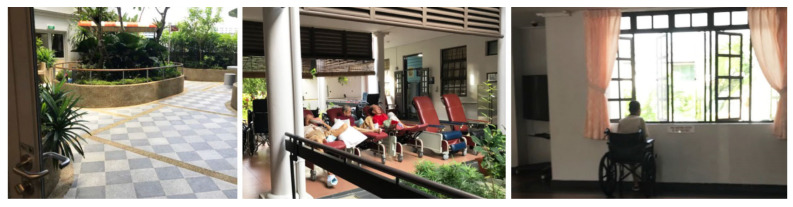
(**Left**) a therapy garden not being used during most of the day in NH3. (**Middle**) residents in geriatric chairs passively sitting in the sun in NH5. (**Right**) a resident in a wheelchair looking outside through the window in NH5.

A majority of the nursing homes in this study had an outdoor space including a garden or a pond. However, these spaces were not well utilized because of access barriers and the hot and humid climate of Singapore. Despite the thermal discomfort, NH5 scheduled “outdoor time”, where residents were lined up in their wheelchairs or geriatric chairs to sit side by side in the heat (Figure 10). While this offers the health benefits of exposure to natural elements, it is unsuitable for use over a longer duration. Similarly, the “therapy garden” in the dementia ward in NH3 was only used for morning exercise. The therapy garden was empty during the rest of the day since it was either too hot or directly exposed to rain. This limited accessibility to outdoor spaces resulted in the residents’ behavior of passively sitting around the window looking outside for extended periods of time.

**Figure 11:**
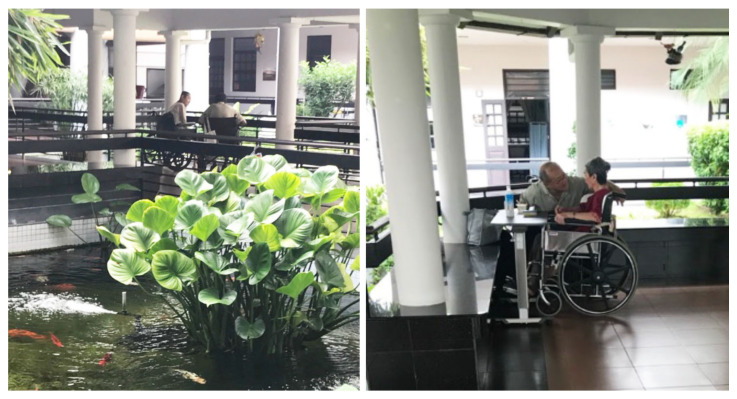
A sheltered gazebo surrounded by a pond, visited by a resident and a family member in NH5.

The design of these outdoor spaces (e.g., lack of canopy trees or sheltering elements) prevented the residents from obtaining the intended benefits. One exception, a well-utilized urban nature space, was the sheltered garden on the ground floor that connected the main lobby to the dining area in NH5 (Figure 11). Access to this space was open—not separated by a door—and featured a wide corridor leading to a seating area. This space was observed to be utilized frequently during physical therapy sessions with residents, family visits and times when residents were casually socializing with each other. As this space was sheltered, it provided shelter from the sun and rain, with a variety of sensory stimulations, including the sound of running water.

### 4.9. Voluntary Creation of Communal Experiences

Some residents were observed voluntarily helping each other. Combining residents with different abilities provided an opportunity for the more able residents to help those who were less so. Residents who were more ambulant helped those with mobility impairments, while residents who were cognitively sound helped those with cognitive impairments during a bingo game.

**Figure 12:**
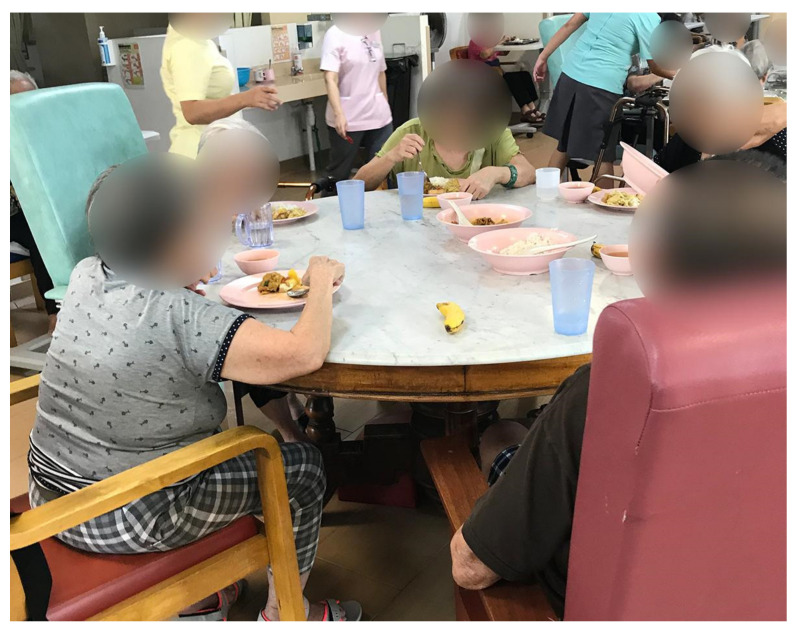
Residents sharing food during a meal in NH4.

The staff in NH4 assigned seating at the dining tables to facilitate relationship building among the residents. The residents formed positive relationships with one another during shared meals, which reflects a practice that resembles the common Singaporean dining culture at home and in public coffee shops and food centers. The food was served in large bowls placed in the middle of the table, and the residents helped themselves instead of being served by the caregiving staff (Figure 12). Peer-to-peer support at the dining table was also observed, as residents helped in serving other residents at their table who needed assistance.

### 4.10. Turning a Family Visit into a Shared Experience

While filial piety is an important value in Singapore, the nursing homes in the case study failed to design spaces with provision for family visits. In most cases, visiting family members found a space at the resident’s bedside or in the large common living room where other residents were also present. There was no designated space for family visits, and the visitors had to bring chairs from the dining room to the resident’s bedside.

**Figure 13:**
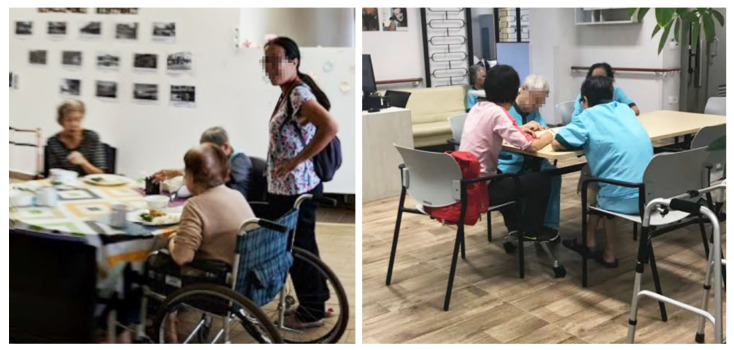
A family member bringing food for the other residents: (**left**) NH1; (**right**) NH3.

Some families accepted the imposed limitations of a shared setting and turned their visits into communal ones. Family members who brought food shared with other residents and helped care for the other residents in the common living spaces (Figure 13). Such acts of turning a problem into an opportunity for sharing are engendered by the culture of collective living that is common in many Asian societies.

## 5. Discussion

Our findings illustrated how the nursing homes in Singapore attempted to implement a sense of home for their residents and how the residents themselves improvised to create—despite limited means—a sense of home through social interactions. In doing so, we identified different criteria for implementing a sense of home, as well as challenges and opportunities in Singapore’s high-density nursing homes. We discuss these in terms of the residents’ management of privacy and territoriality in shared spaces, the need for holistic implementation of homelike environments integrated into building designs and care programs and the pivotal role of communal experiences in fostering a sense of home for the residents.

### 5.1. Privacy Management and Territoriality in the Shared Space

Numerous multidisciplinary studies in long-term care settings have illustrated the important connections between health and well-being and privacy and autonomy in an aging population (e.g., [10,47,48]). Consequently, the prevailing nursing home recommendations from developed economies are for single-bedded rooms to enhance privacy and allow residents to manage their environment [49,50]. Such studies were mostly founded on, and emphasized, the Western-inspired values of neo-liberalism and individualism. Conversely, a few studies in Asia (mostly with an ethnic Chinese profile) have reported an acceptance of multi-bedded rooms in the Asian context [6,7].

Our findings from the study of five Singapore nursing homes corroborate those of Low et al. [7] on older Chinese people in Hong Kong nursing homes, insofar as shared bedrooms are concerned. Since many residents in our study were familiar with communal living and shared bedrooms due to their past lifestyles [10], shared spaces were not seen as an invasion of privacy but as an opportunity to engage in communal experiences.

While the residents in Singapore nursing homes in this study accepted the expectations of communal living, they negotiated a degree of privacy in the shared bedrooms and common living rooms. The small private corners created by furniture and curtains established a semblance of personal space and protected residents from excessive exposure in the shared spaces. The residents’ needs for privacy were found to be especially crucial for the physical aspects of nursing care, and this has also been addressed in multiple studies across different countries (e.g., [13,51,52]).

Furthermore, for the residents in this study, creating a sense of territoriality by using personal objects provided them with some control over the space. This micro-personalization was realized through bedside spaces in shared bedrooms and appropriation of a “regular” seat in common or living spaces. Personalizing the limited space with personal belongings might be the residents’ sole means of mitigating the adverse effects of a low level of privacy on emotional exhaustion, as explained by Laurence et al. [53].

While the residents endeavored to establish a comfortable level of privacy that they felt to be necessary in the shared spaces, the physical design and programmatic and operational concerns of the nursing homes created barriers to such efforts, since many spaces were multi-use (e.g., tables for activities and meals), so that residents were required to set up and remove their personal items several times a day. This reduced the efficacy of their attempts to establish a personal space, and a lack of efficacy has been associated with dysfunctional behaviors [42].

These findings suggest that while multi-bedded rooms are provided in Singapore nursing homes for residents who are familiar with shared environments, the ability to adjust privacy levels and personalization should be carefully enabled. For example, supportive bedside furniture and wall fittings with adequate space for storage and display of personal items should be provided. Residents should be able to adjust the privacy level in shared spaces using adjustable spatial elements such as screens or partitions. In the common rooms, providing defined spaces where residents can leave personal items allows them to establish territoriality and engage in more long-term, stimulating and creative activities.

As the phenomenon of population aging progresses in Singapore, it is also necessary to plan for future generations of older nursing home residents who are better educated and may have different expectations. Hence, to be future-ready, nursing homes must plan for the incorporation of flexible arrangements to accommodate a quantum of single-bedded rooms or double-bedded rooms for couples. Privacy and dignity will continue to remain an enduring yardstick, because they undergird the deep notion of what an authentic homelike environment entails.

Although the overarching mandate of surveillance devalues privacy, it does not mean that safety and privacy are conceptually incompatible. It simply raises the imperative that the design of the environment must include creative solutions. The plethora of readily available technologies (e.g., wearable devices and environmental sensors) provide ample opportunities to advance from an institutional model of nursing homes to one that is less invasive and supports the health and well-being of the residents [54,55,56].

One important aspect of privacy that was neglected is family privacy. Space and time for personal events—such as family visits—were not well supported, especially in terms of physical space provision. Visiting family members often struggled to find a space for private moments with the residents and had to adapt their visit into a shared event by extending it to other residents. While this has its benefits, the trade-off is a loss of private time for familial bonding. As previous studies reported, filial piety primarily concerns a sense of moral responsibility in the traditional Chinese culture [57,58]. This implies that facilitating a family visit to make the experience meaningful and deeply satisfying for both the resident and his/her family should be an important imperative for the future design and management of nursing homes in Singapore.

### 5.2. Holistic Implementation of Homelike Environments

Creating a homelike environment presents many challenges, because such environments encompass many tangible and intangible aspects [59]. While some findings are more prescriptive than others (e.g., single-bedded rooms), the challenge continues to be one of defining what this homelike environment should resemble, as there are no universal norms. Incorporating environmental cues—common household objects, odors, sounds, visual stimuli, etc.—which stimulate reminiscences of the residents’ past life experiences while keeping them oriented to the present are instrumental, yet they must be incorporated in meaningful ways.

Several environmental features in the nursing homes in this study provide examples of how elements can be used to evoke a homelike environment. Murals with familiar scenes, doors that mimic those in HDB estates or the “mama shop”, collectively provided the residents with opportunities to reminisce and promoted a sense of familiarity which linked their previous life to their present one, contributing to their sense of identity [60]. While these measures serve to stimulate the residents’ minds, their location might have reduced their impact. Situating them outside the daily living spaces posed a challenge, as the residents were dependent on the caregiving staff for access to these spaces. Further, independent access to the garden and outdoor spaces was not possible. The location of beneficial environmental features becomes ineffective for improving the residents’ health and well-being when barriers interfere with access.

Furthermore, engaging residents in home-making activities provides an opportunity for them to actively participate in, build an emotional attachment to and foster ownership of their living environment.

### 5.3. Pivotal Role of Communal Experiences

This study revealed how residents voluntarily initiate interactions with fellow residents in their daily routines such as during mealtimes or recreation. This observation is consistent with the postulation by Low et al. [7] that for the older Chinese people in nursing homes, enjoyment is linked to “living together” in a communal living arrangement. This study supports findings indicating the benefit of incorporating design elements that reinforce mutual self-help whenever the opportunity arises and encourage the enduring trait among nursing home residents within the Singapore context. Thus, facilitating a sense of “living together” could play a key role for residents in building a sense of home in Singapore nursing homes, and could be equally as crucial as privacy in shared spaces.

The desired cohesive social compact that was commonly found in traditional *kampongs* (villages) and which was thought be lost to rapid urban renewal was found in the communal practices within the nursing homes in this study. Fung [3] argues that many lessons can be learnt from traditional *kampongs* to create the “down-to-earth simplicity” that is quintessential to Southeast Asian cultures and which pervaded the early communities of Singapore. This re-enchantment with tradition is not simply a result of idle nostalgia but acknowledges that future cohorts of nursing home residents will be naturally predisposed towards mutual self-help, which is an invaluable trait in creating a sense of home.

However, environmental elements for communal behaviors should carefully consider the possibility of overstimulation with negative stimuli, which can interfere with social relationships [13,61]. Most residents in the nursing homes in this case study were negatively affected by moderate-to-severe sensory impairments and excessive environmental stimuli (e.g., noise, glare, visual clutter, thermal discomfort, etc.), which adversely impacted their capacity to adapt to their new environment.

### 5.4. Limitations of the Study

In this observational study, the Hawthorne effect may have been present, given that the residents had provided their consent before joining the study [62]. To minimize adjustment of the residents’ behaviors when being observed in their daily lives, the researchers made multiple visits to each nursing home to socially immerse themselves. In addition, the observations were conducted at a sufficient distance from the residents, so that the presence of the researchers would not cause residents or staff to self-censor their behaviors [63].

While the study participants included multiple ethnic groups, namely Chinese, Malay and Tamil, the majority of the participants were Chinese, which followed the ethnic distribution in each nursing home. One limitation of this study could be that our data did not address whether there were any differences in residents’ experiences depending on their ethnic cultural backgrounds. A future study might investigate this question, to enhance the cultural sensitivity of design [8]. It should also be noted that the findings from this study revealed the social norms and practices particular to Singapore and should be differentiated from the reports on the Chinese ethnic groups in Shanghai [6] or Hong Kong [7]. Our findings add importance to local considerations for the culturally sensitive design of nursing homes, going beyond ethnic stereotyping.

Lastly, the literature data in our study could be strengthened through a systematic literature review. While the review articles referred to in our study revealed the dominance of Anglo-Saxon countries in this field of study (e.g., [1]), the inclusion of studies from other Western areas such as South American or Mediterranean countries would enrich the argument of our study.

## 6. Conclusions

This study highlights different criteria and the extent of implementation of a sense of home in Singapore’s nursing homes, differing from the most commonly featured cases of low-density developments in developed Western countries in the extant literature. Since communities are founded on diverse cultural values and norms, it is important to understand how these intangible aspects and their spatial expressions work within a specific context. The findings from our study support the importance of culturally sensitive designs for creating a genuine sense of home in nursing homes in different environments. The observations in this study are intrinsic to the context in which they were made—the particular time, place and culture—and therefore, generalization to another context will require thoughtful adaptation. The findings from this study reflect current nursing home designs, policies and practices which guide the daily activities and life course experiences of the current cohorts of residents, all of which are constantly evolving.

## Figures and Tables

**Table 1:** Recommendations from the literature review for creating a sense of home in nursing homes.

Themes	Recommendations	Key References
Private spaces	Private rooms allow residents an opportunity to withdraw and be on their own and to create their own environment by having control over space.	Van Hoof et al. [2], Hauge and Heggen [4], Nakrem et al. [5], Eijkelenboom et al. [11], de Veer and Kerkstra [21], van Hoof et al. [19], Falk et al. [22]
	Private rooms allow residents to be able to receive visitors.	Van Hoof et al. [2]
	Access to a private bathroom offers residents a sense of control and privacy during personal care activities.	Fleming et al. [13], van Hoof et al. [19], Klaassens and Meijering [23]
	Furniture in private spaces, meant to accommodate residents’ visitors.	Eijkelenboom et al. [11]
Personal belongings	Residents’ personal belongings such as cherished items and furniture create a semblance of familiarity.	Fleming et al. [13], van Hoof et al. [19], Board and McCormack [24]
	Sufficient space to store and display personal items.	Van Hoof et al. [2]
	Storage space for personal items.	Eijkelenboom et al. [11]
Ambience	Evoke a sense of warmth and coziness through strategies such as having access to daylight, with homelike furniture, decorations and homelike smells.	Van Hoof et al. [2], Eijkelenboom et al. [11], Fleming and Purandare [12], Board and McCormack [24]
	Reserved spaces in communal spaces, such as having their own spot at the dining table, or a private chair in a preferred spot.	Van Hoof et al. [2], Eijkelenboom et al. [11], van Hoof et al. [19]
	Accommodate lower-density spaces within the larger units	Van Hoof et al. [2], Eijkelenboom et al. [11]
	Location in a familiar neighborhood or hometown.	Eijkelenboom et al. [11], Board and McCormack [24]
Appropriate levels of stimulation	Provide activities that promote pleasure and appropriate levels of stimulation, according to the resident’s needs and abilities.	Van Hoof et al. [2], Fleming et al. [13], Fleming and Purandare [12]
	Provide a variety of spaces to suit different functions and preferences (e.g., having quiet spaces to retreat to when someone else is watching TV).	Fleming and Purandare [12]
Connection with nature	Physical and visual access to outdoor and green spaces with fresh air and sun enables connection to the outside world.	Van Hoof et al. [2], Nakrem et al. [5], van Hoof et al. [19]
	Animal life (e.g., birds or fish) provides a welcomed distraction for residents.	Van Hoof et al. [2], van Hoof et al. [19]
Social interactions	Supporting engagement in everyday homelike activities invites residents and their families to be involved in everyday life.	Robinson et al. [16], van Zadelhoff et al. [18]
	Comfortable spaces conducive to engagement and interaction with other residents, family and visitors.	Van Hoof et al. [2], Falk et al. [22]

**Table 2:** Profiles of the five nursing homes in terms of building and resident types.

	NH1	NH2	NH3	NH4	NH5
Site area (sqm)/Number of stories	4830/ 11 stories	2800/ 12 stories	3000/ 9 stories	2991/ 6 stories	9056/ 3 buildings: (1), (2) and (3), each 3 stories high
Bedroom type	4 beds	9 beds and 4 beds	8 beds	6 beds	(1) 8 beds (2) 5 beds (3) 2 beds
Typical bedroom size (sqm)	36	85	64	90	(1) 54 (2) 50 (3) 20
Beds occupied/ Total capacity	343/392	560/600	203/225	218/230	190/190
Types of residents *	Cat I: 0 Cat II: 27 Cat III: 198 Cat IV: 118	Cat I: 0 Cat II: 14 Cat III: 318 Cat IV: 228	Cat I: 0 Cat II: 5 Cat III: 103 Cat IV: 95	Cat I: 1 Cat II: 6 Cat III: 83 Cat IV: 128	Cat I: 0 Cat II: 0 Cat III: 128 Cat IV: 162
Ethnicity of residents	Chinese: 87.1% Malay: 8.9% Indian: 4% Others: 0%	Chinese:96% Malay: 0% Indian: 4% Others: 0%	Chinese: 61.3% Malay: 24.7% Indian: 12.9% Others: 1.1%	Chinese: 88% Malay: 4% Indian: 8% Others: 0%	Chinese: 60% Malay: 10% Indian: 24% Others: 6%
Unit type	Household: 4 rooms × 4 beds	Ward-wing: 4 rooms × 8 beds (plus 1 room × 4 beds (haze room))	Ward-floor: 6 rooms × 8 beds	Ward-floor: 9 rooms × 6 beds	Ward-floor cluster: (1) 8 rooms × 8 beds (2) 6 rooms × 5 beds (3) 8 rooms × 2 beds
Residents per unit	16	36	48	54	(1) 64 (2) 30 (3) 16
Staff per unit	5 (plus 1 shared staff member between units)	15 to 17	10	18 to 20	60

* The resident’s functional status is assessed using the Residential Assessment Form (RAF), which comprises nine indicators with a point-scoring system to categorize residents from 1 to 4, defined as follows: Category I: ambulant, able to perform activities of daily living (ADL) independently; Category II: semi-ambulant, able to perform ADL semi-independently; Category III: non-ambulant, requires assistance with ADL and wheelchair-bound; Category IV: bedbound, dependent on caregiver for ADL, requires medical and nursing care.

**Table 3:** Profiles of the study participants (residents) from the five nursing homes.

		NH1		NH2		NH3		NH4		NH5	
**Total Residents Recruited**		101	29.5%	99	17.7%	95	46.8%	100	45.9%	100	52.6%
**Participant’s age group**	**<40**	0	0%	0	0%	1	1.1%	0	0%	0	0%
	**40–50**	3	3%	1	1%	3	3.2%	1	1%	3	3%
	**51–60**	7	6.9%	2	%	12	12.6%	2	2%	13	13%
	**61–70**	17	16.8%	22	22.2%	18	19%	17	17%	18	18%
	**71–80**	31	32.7%	26	42.4%	29	28.4%	33	31%	27	32%
	**81–90**	33	32.7%	42	42.4%	27	28.4%	31	31%	32	32%
	**91–100**	9	8.9%	6	6.1%	5	5.3%	12	12%	7	7%
	**>100**	1	1%	0	0%	0	0%	2	2%	0	0%
	**Unknown**	0	0%	0	0%	0	0%	2	2%	0	0%
**Participant’s length of stay** **(years)**	**0**	19	18.8%	35	35.4%	65	68.4%	20	20%	40	40%
	**1–3**	50	49.5%	37	37.4%	29	30.5%	37	37%	27	27%
	**4–6**	23	22.8%	18	18.2%	0	0%	18	18%	15	15%
	**7–10**	5	5%	8	8.1%	0	0%	11	11%	9	9%
	**>10**	4	4%	1	1%	0	0%	11	11%	9	9%
	**No data**	0	0%	0	0%	1	1.1%	3	3%	0	0%
**Participant’s RAF category**	**Cat II**	7	6.9%	2	2%	5	5.3%	0	0%	0	0%
	**Cat III**	62	61.4%	80	80.8%	66	69.5%	41	41%	29	29%
	**Cat IV**	32	31.7%	14	14.1%	24	25.3%	59	59%	71	71%
**Participants ethnicity**	**Chinese**	88	87.1%	95	96%	59	62.1%	88	88%	60	60%
	**Malay**	9	8.9%	0	0%	23	24.2%	4	4%	24	24%
	**Indian**	4	4%	4	4%	12	12.6%	8	8%	10	10%
	**Others**	0	0%	0	0%	1	1.1%	0	0%	6	6%
**Participants gender**	**Male**	61	60.4%	44	44.4%	53	55.8%	48	48%	54	54%
	**Female**	40	39.6%	55	55.6%	42	44.2%	52	52%	46	46%
**Diagnosed with dementia**	**Yes**	42	41.6%	61	61.6%	28	29.5%	56	56%	59	59%
	**No**	59	58.4%	38	38.4%	67	70.5%	44	44%	41	41%
**Ability to communicate**	**Yes**	93	92.1%	95	96%	93	97.9%	86	86%	100	100%
	**No**	8	7.9%	4	4%	2	2.1%	14	14%	0	0%
